# Supplementary material for: Comparative Colorimetric Sensor Based on Bi-Phase γ-/α-Fe2O3 and γ-/α-Fe2O3/ZnO Nanoparticles for Lactate Detection
Source: Biosensors (Basel). 2022 Nov 16;12(11):1025. doi: 10.3390/bios12111025 (PMC9688618; doi:10.3390/bios12111025)
Supplement: Supplementary file 1 [file biosensors-12-01025-s001.zip › biosensors-1994326-supplementary.pdf]

biosensors

Supplementary Materials

Comparative Colorimetric Sensor Based on Bi-phase γ-/α-Fe_2O_3 and γ-/α-Fe_2O_3/ZnO Nanoparticles for Lactate Detection

Ricardo A. Escalona-Villalpando [1], Karen. Viveros-Palma [1], Fabiola I. Espinosa-Lagunes [2], José A. Rodríguez-Morales [1], Luis G. Arriaga [2], Florika C. Macazo [3], Shelley D. Minteer [3] and Janet Ledesma-García [1,*]

[1] División de Investigación y Posgrado, Facultad de Ingeniería, Universidad Autónoma de Querétaro, 76010, Santiago de Querétaro, México
[2] Centro de Investigación y Desarrollo Tecnológico en Electroquímica, Pedro Escobedo 76703, Querétaro, México.
[3] Department of Chemistry, University of Utah, 315 South 1400 East, Salt Lake City, UT 84112, USA.

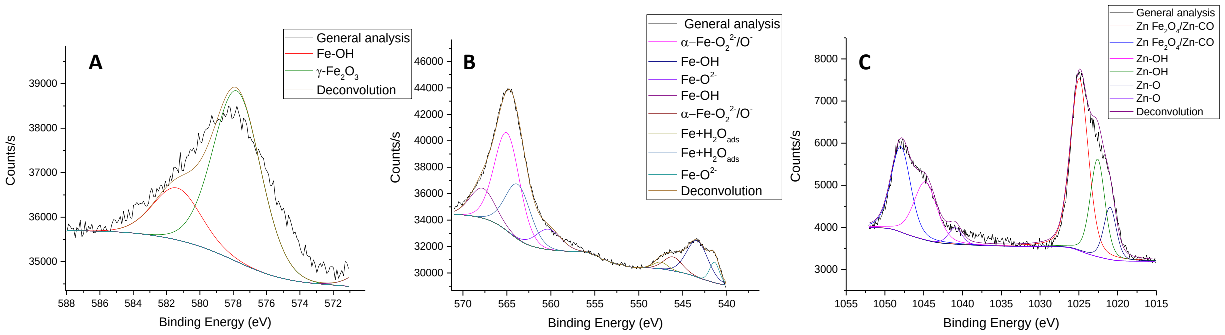

Figure S1. XPS spectra and corresponding fitting curves of γ-Fe_2O_3 (A), α-Fe_2O_3 (B), and ZnO (C)..

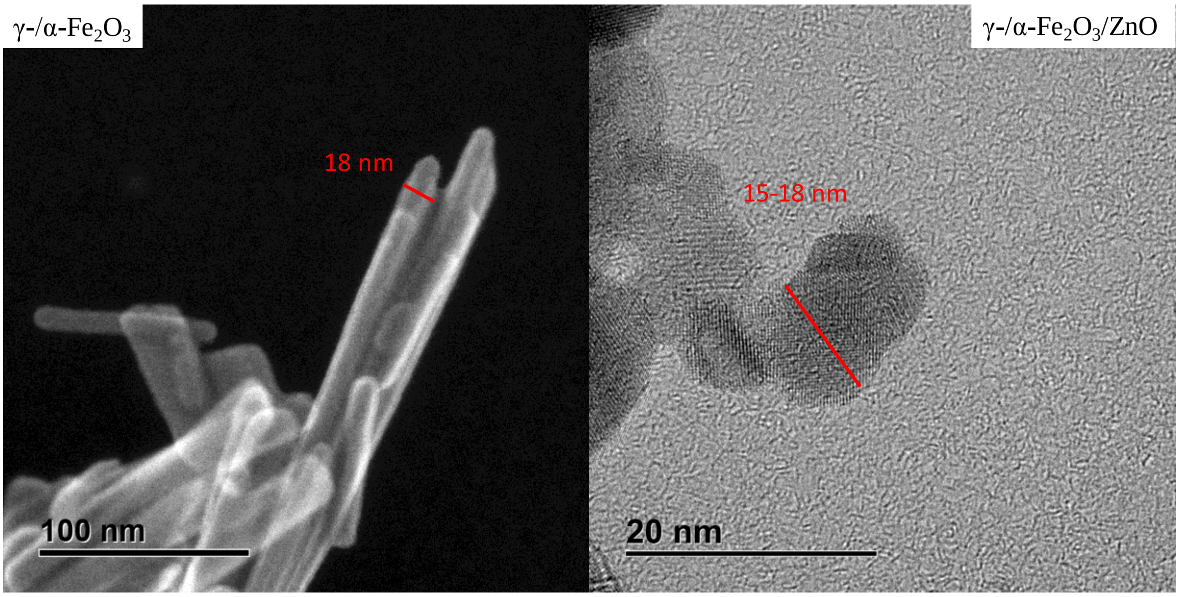

Figure S2. S/TEM images of the size of γ-/α-Fe_2O_3 (left) and γ-/α-Fe_2O_3/ZnO composite particles (right).

The determination of the working wavelength was through the reaction performed in 1.9 mL NaHOAc buffer (0.01 M, pH 4.0), 30 µL of 500 µM TMB, 30 µL H_2O_2 at 30% and 20 µL NPs (5 mg mL^{-1}). The wavelength change was made every 20 nm between 400 - 800 nm and the measurement was made after 10 min of leaving the reagents in equilibrium.

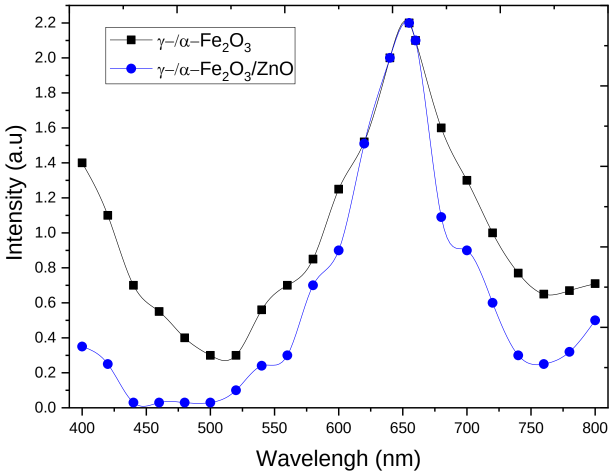

Figure S3. Absorbance spectra of TMB–H_2O_2 of γ-/α-Fe_2O_3 (black line) and γ-/α-Fe_2O_3/ZnO (blue line) solutions. The reaction was performed in 1.9 mL NaHOAc buffer (0.01 M, pH 4.0), 30 μL of 500 μM TMB, 30 μL H_2O_2 at 30% and 20 μL NPs (5 mg mL^{-1}).

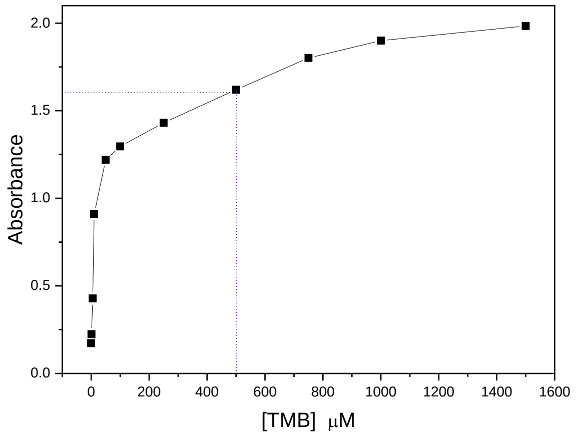

Figure S4. Plot of absorbance as a function of the concentration of TMB with 1 mM of H_2O_2.

Table S1. Zn/Fe/O and γ-Fe_2O_3:α-Fe_2O_3 ratio from γ-/α-Fe_2O_3/ZnO.

	**Fe_2O_3**		**Fe_2O_3 ZnO**		
	Fe/O	O/Fe	Fe/O	Fe/Zn	Zn/O
**Weight %**	0.046	21.49	1.05	0.48	2.19
**Pp, Atom%**	2.38	0.42	0.14	0.23	4.25

	**γ-Fe_2O_3**	**α-Fe_2O_3**
	Fe/O	Fe/O
**Pp, Atom%**	1.64	1.48

The Samples were analyzed in the monochromatic equipment magics thermo scientifics, model k-alpha (XPS). An area analysis was performed, selecting three sections, for each element present (Faith, O and Zn respectively), different scanning times were used, to obtain more defined spectra. Surface Analysis software was released for the S1 table (tables are appended from the analyses).

The Fe/O (was made in all samples), Fe/Zn and Zn/O ratios (for the sample containing Zn) is displayed, considering the % by weight and the atomic % provided by the analysis when making the deconvolutions (adjusting the results). For the % by weight the value was taken from the general analysis and for it atomic % took the values of the sweeps, since in each scan it penetrates more into the sample (so you can see more interactions at the atomic level).
